# Prospective cohort of parameters of glycemic and lipid metabolism after abdominoplasty in normal weight and formerly obese patiens

**DOI:** 10.1016/j.jpra.2023.07.005

**Published:** 2023-07-17

**Authors:** Leandro R.de A. Santos, Paulo R. da Costa, Thiago S. Maia, Armando Chiari Junior, Vivian Resende

**Affiliations:** a Master in Sciences Applied to Surgery and Ophtalmology graduate program of the Medical School, Federal University of Minas Gerais, Belo Horizonte, Brazil; b Surgery Department of the Medical School, Federal University of Minas Gerais, Belo Horizonte, Brazil; c Graduated at Medical School Federal, University of Minas Gerais, Belo Horizonte, Brazil; d Surgery Department of the Medical School and Head of the Plastic Surgery service at Clinical Hospital, Federal University of Minas Gerais, Belo Horizonte, Brazil; e Surgery Department and Coordinator of the Sciences Applied to Surgery and Ophtalmology graduate program of the Medical School, Federal University of Minas Gerais, Belo Horizonte, Brazil

**Keywords:** Obesity, Abdominoplasty, Body contouring, Metabolism, Carbohydrate metabolism, Lipid metabolism

## Abstract

**Background:**

Obesity poses a major risk for cardiovascular diseases, while it is almost a consensus that intra-abdominal adiposity has a more deleterious effect for metabolic syndrome. In this sense, it is speculated that lipectomy or liposuction would be metabolically harmful, as it changes the abdominal-superficial adipose tissue ratio. However, the literature has shown conflicting evidence.

**Methods:**

In order to evaluate the possibility of metabolism alteration resulting from body coutouring surgery, a prospective cohort was implemented with 35 patients who underwent abdominoplasty, including some with a history of massive weight loss. Fasting blood glucose, fasting plasma insulin, triglycerides, total cholesterol and fractions were requested preoperatively and in the third postoperative month. The groups were also compared with each other.

**Results:**

No statistically significant variation between the exams collected in the preoperative period and those collected after abdominoplasty was found. There was a statistically significant difference in LDL (low-density lipoprotein; *p* = 0.033) and non-HDL (non-high-density lipoprotein) cholesterol (*p* = 0.020) between the two control tests of the groups surveyed. There were also differences in comorbidities (*p* = 0.006) and complications (*p* <0.001) between the groups.

**Conclusions:**

Abdominoplasty was not able of changing tests that assess glycemic and lipid metabolism three months after the operation. Our attention was drawn to the fact that patients who had massive weight loss had better control of LDL cholesterol (*p* = 0.033) and non-HDL cholesterol (*p* = 0.020), despite having higher weight and body mass index (*p* <0.001).

## Introduction

One billion and seven hundred million people are classified as obese and predictions based on a linear time trend suggest that 51% of the United States population will also be by 2030.^1^^,^^2^ Obesity is defined as a body mass index (BMI) of greater than 30 kg/m^2^. It poses a major risk for cardiovascular disease (CVD), type 2 diabetes (T2DM), hypertension, stroke, certain types of cancer and mortality. Abdominal obesity (upper-body type of fat distribution or apple-shaped), along with elevated serum triglycerides, low HDL (high-density lipoprotein) cholesterol, elevated blood pressure, insulin resistance, and high rates of atherosclerotic disease, is considered a component of the metabolic syndrome (MetS).^1^^,^^3^, ^4^, ^5^ Currently, approximately one third of the adult world population suffers from MetS, having increased risk for the development of T2DM and CVD.¹

Obesity stems mainly from excess food calories, which is stored in the form of triglycerides in adipose tissue. It is known that this tissue is not inert, and that it is subdivided into visceral and subcutaneous compartments. There is a hormonal and immunological function, evidenced by interleucin-6 (IL-6) secretions, especially by visceral adipocytes, and adiponectin and leptin, which are greater in subcutaneous tissue.^6^^,^^7^ It is almost a consensus that intra-abdominal adiposity has a more deleterious effect for MetS. In this sense, it can be speculated that lipectomy or liposuction would be metabolically harmful, as it changes the abdominal-superficial adipose tissue ratio.^3^ However, the literature has shown conflicting evidence, regarding blood pressure, triglycerides, insulin concentrations and insulin sensitivity in the short-term.¹

These uncertainties in papers that have already been published, the increasing performance of esthetic procedures, and the epidemiology of obesity with its enormous biopsychosocial impact, justify the realization of new research in the area. Based upon this and in order to evaluate the possibility of metabolism alteration, we followed 35 patients who underwent abdominoplasty, including some with a history of massive weight loss (MWL - defined as weight loss greater than or equal to 22 kg). Abdominal dermolipectomy was chosen because it is usually the body contouring plastic surgery with the largest tissue resection.

## Materials/patients and methods

A prospective cohort was implemented with 35 patients. This research was approved by the Ethics Committees at Federal University of Minas Gerais under the protocol number 2.334.697. Free and informed consent terms were filled in by all participants. Two groups were considered in the study:

• Group 1: 16 patients who lost at least 22 kg and underwent anchor abdominoplasty;
• Group 2: 19 patients without MWL in which classic abdominoplasty was performed.

The following laboratory tests were requested twice for all individuals: fasting blood glucose, fasting plasma insulin, triglycerides, total cholesterol and fractions. The exams were collected in the Clinical Hospital laboratory and methods were: chemiluminescence for insulin; colorimetric for blood glucose, triglycerides, total and HDL cholesterol; end point for LDL (low-density lipoprotein) cholesterol; and calculation for non-HDL and VLDL (very low-density lipoprotein) fractions. The first dosage was taken preoperatively and the second dosage was performed in the third postoperative month of plastic surgery. This time period was determined in order to avoid essentialy two bias: (1) the inflammatory response to trauma in the initial postoperative period; (2) the lifestyle change by performing physical exercises after the convalescence period. The three-month exams were compared with preoperative exams, as we compared the control exams between the groups. Other data collected for comparison were comorbidities, complications, age, weight, BMI, amount of resected tissue and liposuction volume. In fact, conventional liposuction in the anterior abdomen and flanks was associated in two patients of group 1 and in 17 of group.

People with diabetes mellitus, whose abdominoplasty was secondary or merely hygienic, or groups of isolated liposuction or abdominoplasty associated with back liposuction, were not included in the study. Altogether, two individuals who would be in group 1 and five who would be in group 2 were excluded, adding up to the aforementioned 16 and 19 people, respectively. The reason for these exclusions was the failure to repeat the requested exams.

Statistical calculation was done using SPSS software (IBM SPSS, Chicago, IL, version 25). Wilcoxon test was used for paired exams, Mann-Whitney U test for comparison of control exams in each group, and Fisher's exact test to assess if there were differences between comorbidities and complications between the groups. Significance was set at *p* < 0.05 and study size was arrived based on previous literature review.

## Results

There was no statistically significant variation between the exams collected in the preoperative period and those collected in the third month after abdominoplasty, whether after massive weight loss or not (Tables 1 and 2).

**Table 1:** Statistics regarding laboratory tests of the Group 1 patients with MWL.

	First dosage media	Second dosage media	*p*-value
Fasting blood glucose	87 mg/dl	87 mg/dl	0.795
Fasting plasma insulin	4 mg/dl	5 mg/dl	0.374
Triglycerides	81 mg/dl	90 mg/dl	0.162
Total colestherol	174 mg/dl	187 mg/dl	0.112
HDL	61 mg/dl	63 mg/dl	0.299
LDL	97 mg/dl	105 mg/dl	0.326
Non-HDL	113 mg/dl	124 mg/dl	0.125
VLDL	16 mg/dl	18 mg/dl	0.091

**Table 2:** Statistics regarding laboratory tests of the Group 2 patients, who underwent classic abdominoplasty.

	First dosage media	Second dosage media	*p-*value
Fasting blood glucose	87 mg/dl	87 mg/dl	0.641
Fasting plasma insulin	6 microU/ml	7 microU/ml	0.375
Triglycerides	127 mg/dl	133 mg/dl	0.165
Total colestherol	203 mg/dl	207 mg/dl	0.702
HDL	53 mg/dl	57 mg/dl	0.324
LDL	124 mg/dl	124 mg/dl	0.231
Non-HDL	150 mg/dl	150 mg/dl	0.520
VLDL	22 mg/dl	27 mg/dl	0.064

There was a tendency to increase the VLDL in both groups, with *p* = 0.091 in group 1 and *p* = 0.064 in group 2. However, as stated, this was not enough to compose significance**.**

The most intriguing finding of the study was the statistically significant difference in LDL and non-HDL cholesterol between the two control tests of the groups surveyed (Table 3). Prior to surgery, mean LDL in Group 1 was 97 mg/dl, while the average for Group 2 was 124 mg/dl. Mean values ​​of non-HDL cholesterol were, respectively: 113 mg/dl and 150 mg/dl. Thus, patients who lost at least 22 kg had lower LDL and non-HDL cholesterol values, with respective p-values ​​of 0.033 and 0.020.

**Table 3:** Statistics comparing preoperative tests between patients of Group 1 (MWL) and Group 2 (who underwent classic abdominoplasty).

	Group 1 first dosage media	Group 2 first dosage media	*p*-value
Fasting Blood glucose	87 mg/dl	87 mg/dl	0.756
Fasting Plasma insulin	4 microU/ml	6 microU/ml	0.142
Triglycerides	81 mg/dl	127 mg/dl	0.257
Total colestherol	174 mg/dl	203 mg/dl	0.056
HDL	61 mg/dl	53 mg/dl	0.182
*LDL*	***97*** ***mg/dl***	***124*** ***mg/dl***	***0.033***
*Non-HDL*	***113*** ***mg/dl***	***150*** ***mg/dl***	***0.020***
VLDL	16 mg/dl	22 mg/dl	0.347

On the other hand, there was no statistical difference in mean age (47 and 43 years, respectively for Groups 1 and 2; *p* = 0.317) and in the other exams surveyed: fasting glucose, fasting insulin, triglycerides, total cholesterol, VLDL and HDL.

Weight and BMI were also compared. Group 1 averages before abdominoplasty were respectively 78.325 kg and 29.2 kg/m², while these values for Group 2 were 63.4474 kg and 24.6574 kg/m². *p*-values ​​for these variables were less than 0.001, what shows a significant difference between the groups.

**Figure 1:**
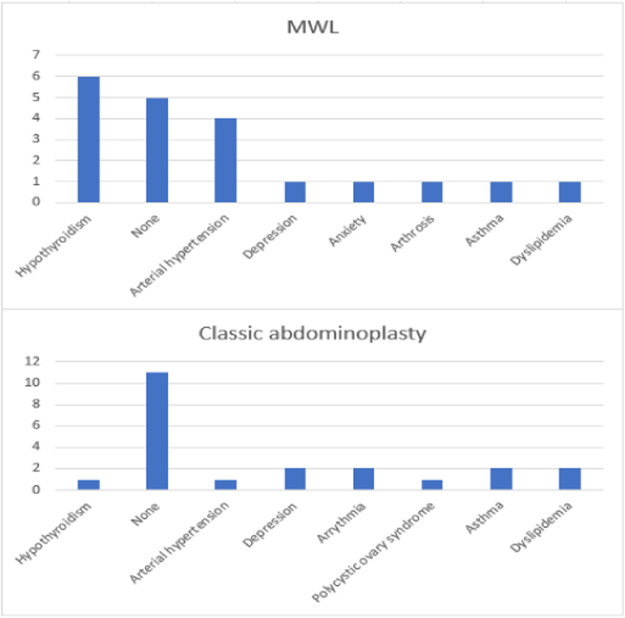
Column charts with the comorbidities of each group.

**Figure 2:**
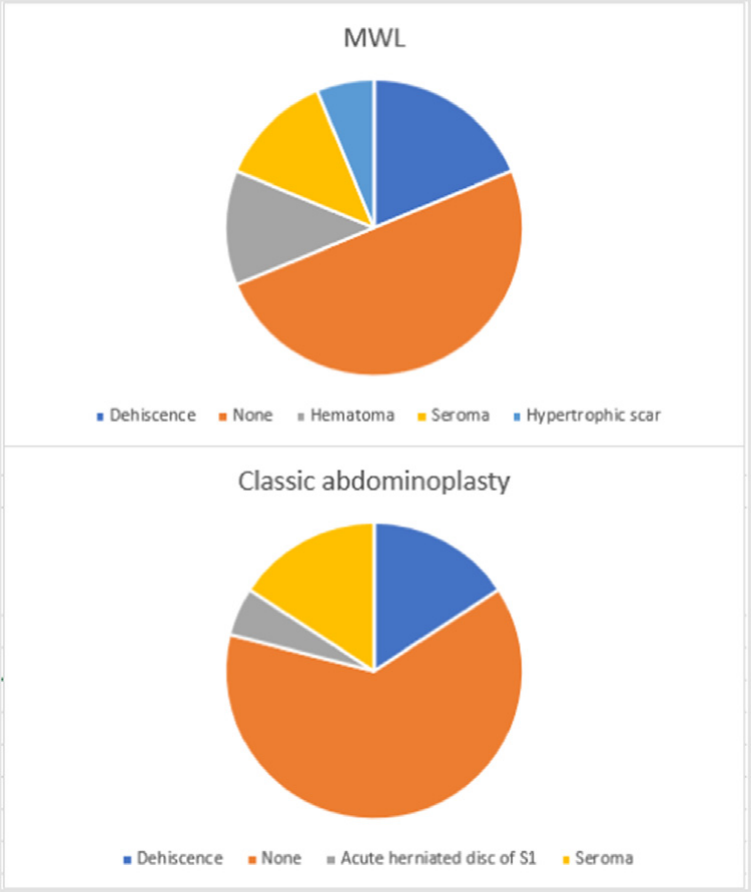
Pie charts with complications after abdominoplasty in each group.

Other differences were comorbidities (*p* = 0.006; Figure 1) and complications (*p <* 0.001; Figure 2). Patients in Group 2 had a higher prevalence of no comorbidity, arrhythmia, asthma, depression, dyslipidemia and polycystic ovary syndrome. Group 1 individuals were more hypertensive and hypothyroid. In addition, Group 1 had higher rates of hematoma, hypertrophic scar and dehiscence, compared to more seroma or no complications in Group 2.

Furthermore, it is noteworthy that the average amount of resected tissue was 2725.625 g in patients of Group 1 and 644.74 g in Group 2. The mean liposuction volume was respectively 18.75 ml and 1024.21 ml.

## Discussion

The absence of statistically significant differences in the metabolic parameters surveyed after three months of performing abdominoplasty in our study is congruent with most of medical literature.

Examples are reviews by Payer et al., Sailor et al. and Danilla et al.,^3^^,^^5^^,^^8^ which involved liposuction, and by Seretis et al. and Marcadenti et al., who evaluated tissue resections.^1^^,^^7^ Moreover, no improvement of insulin sensitivity were found by Fabbrini et al., who undertook omentectomy in 22 obese humans.^9^

Regarding the obese population, Cuomo et al. followed 128 patients after a year of abdominoplasty and have also found no differences in metabolic parameters.^10^

Hernandez et al. randomized 32 nonobese patients into liposuction and control groups. No effect on metabolic markers was found after six months or one year.^11^

Likewise, the work of Lubkowska A. and Chudecka M. with a gluteal-femoral liposuction in women with normative weight showed no changes in the lipid profile.^12^

Unlike most of these works and our findings, reviews by Benatti et al. and Boriani et al. showed significant improvement in insulin sensitivity after liposuction in individuals with overweight or mild obesity.^6^^,^^4^ The use of VASER (amplification of sound energy vibration in resonance) ultrasonic liposuction also presented to be able to attenuate some degree of insulin resistance after four months postoperatively.^13^ Meanwhile, Modolin et al. found interleukins and glucose fall after 14 days of post-bariatric abdominoplasty, however it is notorious that this follow-up is quite short.^14^

Cintra et al. evaluated metabolic and inflammatory parameters in 40 post-bariatric patients, 20 of whom underwent abdominoplasty and the other half comprised of a mastopexy control group. Subsequent long monitoring indicated an increase in HDL cholesterol and a descrease in C-reactive protein after abdominoplasty.^15^

Swanson, in a prospective study with 322 individuals with a mean BMI of 26.6 kg/m², identified a 43% reduction in triglycerides after three months of liposuction in patients with an initial value greater than or equal to 150 mg/dl. This difference was not found in patients without dyslipidemia.^16^ Differently, a study on cryolipolysis showed that this modality can lead to hypertriglyceridemia.^17^

We may wonder whether our results could have been different if the studied population was mostly dyslipidemic or still obese. The main reason for not including obese patients in our work is that this population is generally not ideal for performing plastic surgery. Another consideration is that most of the previous works only contemplate liposuction. New studies involving lipectomies and/or with dyslipidemic individuals are essential to determine if there is any change in metabolism as a result of abdominoplasty.

However, if we did not find metabolic benefits after the surgeries performed, no worsening that could impact a greater tendency to CVD was also detected. As a matter of fact, attempts to mitigate body contouring surgeries are not uncommon, claiming a deleterious increase in abdominal-superficial adipose tissue ratio or using reasoning such as the lipostatic theory proposed by Kennedy, in which the long-term energy balance is achieved through the feedback systems that constantly regulate adipose tissue depots.^18^ These theories seem quite applicable to rats, but the same cannot easily be said for complex human beings.

We already expected that the two groups would be very different in relation to the resected tissue and its complications, and that patients would present another profile of comorbidities despite a similar age. The complication rate of reconstructive surgery after MWL is described as until 31–66%. Healing is affected in 43% of cases, according to systematic review by Albino et al., and these rates are higher than those of transplanted and burned patients and lower than those with neoplasia. There are several explanations for this, one of which is the desregulation of the levels of intercellular matrix metalloproteinases and tissue inhibitors of these proteins.^19^

The most surprising findings were the significant differences in LDL and non-HDL cholesterol between group 1 and group 2, with worse control in those patients with ideal weight. We developed two hypotheses to explain this: (1) the patient who presented a MWL already had his/her metabolism controlled, which is attested by several studies that show metabolic benefits from gastroplasty; (2) the patient who seeks the plastic surgeon to perform a lipoabdominoplasty to treat localized fat has a greater tendency to dyslipidemia. What reinforces this second line of reasoning is the mean LDL value found equal to 124 mg/dl, that would already indicate a change in lifestyle or drug therapy depending on cardiovascular risk, and the mean weight of group 2, that was lower than group 1’s. So, higher levels of LDL and non-HDL cholesterol could not be attributed to overweight or obesity.

Perhaps the most plausible is a mix of these two possibilities. Therefore, the patient with MWL had metabolic benefits from his/her weight loss, and the patient with lipodystrophy and is of normal weight does not follow a balanced diet or exercise plan that allows good lipid control.

If we assume that all of this is true, how important are these findings? Must plastic surgeons recommend physical activities in the postoperative period of their patients? Should the lipid profile be requested before and after body contouring surgery? If any changes are found, might treatment with statins be started? There are many questions that can be extrapolated from this observation, and certainly more researches are needed to answer them and to confirm what we found.

This study was not without limitations. Certainly, the relatively small group of subjects can be included among them. At the same time, more distant effects than three months could be considered. There is also the difficulty in standardizing the patients' diet in the postoperative period, which would be ideal. Also, we could have done other dosages like adiponectin and leptin. But we can not fail to mention that a merit was to involve the post-bariatric population, as there are few studies that assess metabolic alteration after plastic surgery in these individuals, who are increasingly present in our office.

## Conclusions

Classical abdominoplasty is completely different from the anchor type in the patient after MWL, both in relation to tissue resection, as well as complications (*p <* 0.001) and patients' comorbidities (*p <* 0.001). Even so, none of these procedures was able to change, with statistical significance, the tests that assess glycemic and lipid metabolism three months after the operation. Our attention was drawn to the fact that patients who had MWL had better control of LDL cholesterol (*p* = 0.033) and non-HDL cholesterol (*p* = 0.020), despite having higher weight and BMI (*p <* 0.001). Perhaps, these metabolic differences suggest that patients who want treatment for localized adiposity have a tendency to have dyslipidemia. Further studies are in need to evaluate this hypothesis and what it could change in clinical practice.

## Ethical approval

This research was approved by the Ethics Committees at Federal University of Minas Gerais under the protocol number 2.334.697

## Declaration of Competing Interest

None.

## References

[1] Seretis K., Goulis D.G., Koliakos G., Demiri E. (2015). The effects of abdominal lipectomy in metabolic syndrome components and insulin sensitivity in females: A systematic review and meta-analysis. Metabolism.

[2] Hurwitz D.J., Ayeni O. (2016). Body contouring surgery in the massive weight loss patient. Surg Clin North Am.

[3] Payer J., Ziak P., Fedeles J., Brazdilova K., Fedeles J. (2013). Changes in metabolic syndrome parameters after liposuction. Bratisl Lek Listy.

[4] Boriani F., Villani R., Morselli P.G. (2014). Metabolic effects of large-volume liposuction for obese healthy women: a meta-analysis of fasting insulin levels. Aesthetic Plast Surg.

[5] Sailon A.M., Wasserburg J.R., Kling R.R., Pasick C.M., Taub P.J. (2017). Influence of large-volume liposuction on metabolic and cardiovascular health: A systematic review. Ann Plast Surg.

[6] Benatti F.B., Lira F.S., Oyama L.M., do Nascimento C.M., Lancha A.H. (2011). Strategies for reducing body fat mass: Effects of liposuction and exercise on cardiovascular risk factors and adiposity. Diabetes Metab Syndr Obes.

[7] Marcadenti A., de Abreu-Silva E.O. (2015). Different adipose tissue depots: Metabolic implications and effects of surgical removal. Endocrinol Nutr.

[8] Danilla S., Longton C., Valenzuela K. (2013). Suction-assisted lipectomy fails to improve cardiovascular metabolic markers of disease: A meta-analysis. J Plast Reconstr Aesthet Surg.

[9] Fabbrini E., Tamboli R.A., Magkos F. (2010). Surgical removal of omental fat does not improve insulin sensitivity and cardiovascular risk factors in obese adults. Gastroenterology.

[10] Cuomo R., Russo F., Sisti A. (2015). Abdominoplasty in mildly obese patients (BMI 30-35kg/m2): Metabolic, biochemical and complication analysis at one year. In Vivo (Brooklyn).

[11] Hernandez T.L., Kittelson J.M., Law C.K. (2011). Fat redistribution following suction lipectomy: Defense of body fat and patterns of restoration. Obesity (Silver Spring).

[12] Lubkowska A., Chudecka M. (2019). The effects of small-volume liposuction surgery of subcutaneous adipose tissue in the gluteal-femoral region on selected biochemical parameters. Int J Environ Res Public Health.

[13] Gibas-Dorna M., Szulińska M., Turkowski P. (2017). The effect of VASER abdominal liposuction on metabolic profile in overweight males. Am J Mens Health.

[14] Modolin M.L.A., Cintra W., Rocha R.I. (2019). Analysis of inflammatory and metabolic biomarkers in patients submitted to abdominoplasty after bariatric surgery. Acta Cir Bras.

[15] Cintra W., Modolin M., Faintuch J., Gemperli R., Ferreira M.C. (2012). C-reactive protein decrease after postbariatric abdominoplasty. Inflammation.

[16] Swanson E. (2011). Prospective clinical study reveals significant reduction in triglyceride level and white blood cell count after liposuction and abdominoplasty and no change in cholesterol levels. Plast Reconstr Surg.

[17] Klein K.B., Bachelor E.P., Becker E.V., Bowes L.E. (2017). Multiple same day cryolipolysis treatments for the reduction of subcutaneous fat are safe and do not affect serum lipid levels or liver function tests. Lasers Surg Med.

[18] Benatti F., Solis M., Artioli G. (2012). Liposuction induces a compensatory increase of visceral fat which is effectively counteracted by physical activity: A randomized trial. J Clin Endocrinol Metab.

[19] Albino F.P., Koltz P.F., Gusenoff J.A. (2009). A comparative analysis and systematic review of the wound-healing milieu: Implications for body contouring after massive weight loss. Plast Reconstr Surg.

